# Laser-Induced Breakdown Spectroscopy Associated with the Design of Experiments and Machine Learning for Discrimination of *Brachiaria brizantha* Seed Vigor

**DOI:** 10.3390/s22145067

**Published:** 2022-07-06

**Authors:** Guilherme Cioccia, Carla Pereira de Morais, Diego Victor Babos, Débora Marcondes Bastos Pereira Milori, Charline Z. Alves, Cícero Cena, Gustavo Nicolodelli, Bruno S. Marangoni

**Affiliations:** 1 SISFOTON-UFMS—Laboratório de Óptica e Fotônica, UFMS—Universidade Federal de Mato Grosso do Sul, Campo Grande 79070-900, MS, Brazil; guilherme.cioccia@ufms.br (G.C.); cicero.cena@ufms.br (C.C.); 2 Embrapa Instrumentation, São Carlos 13560-970, SP, Brazil; moraispcarla@gmail.com (C.P.d.M.); diegobabos@hotmail.com (D.V.B.); debora.milori@embrapa.br (D.M.B.P.M.); 3 Programa de Pós-Graduação em Agronomia, UFMS—Universidade Federal de Mato Grosso do Sul, Chapadao do Sul 79560-000, MS, Brazil; charline.alves@ufms.br; 4 Departamento de Física, Universidade Federal de Santa Catarina, Florianópolis 88020-302, SC, Brazil; gustavo.nicolodelli@ufsc.br

**Keywords:** LIBS, machine learning, design of experiments, discriminating, brachiaria seed

## Abstract

Laser-induced breakdown spectroscopy (LIBS) associated with machine learning algorithms (ML) was used to evaluate the *Brachiaria* seed physiological quality by discriminating the high and low vigor seeds. A 2^3^ factorial design was used to optimize the LIBS experimental parameters for spectral analysis. A total of 120 samples from two distinct cultivars of *Brachiaria brizantha* seeds exhibiting high vigor (HV) and low vigor (LV) in standard tests were studied. The raw LIBS spectra were normalized and submitted to outlier verification, previously to the reduction data dimensionality from principal component analysis. Supervised machine learning algorithm parameters were chosen by leave-one-out cross-validation in the test samples, and it was tested by external validation using a new set of data. The overall accuracy in external validation achieved 100% for HV and LV discrimination, regardless of the cultivar or the classification algorithm.

## 1. Introduction

High performance in the germination process, plant growth, and plant population in the field is associated with the physiological quality of the seeds [1,2,3], which can be classified as vigor. Vigor summarizes the sum of seed characteristics directly related to germination rates and uniformity, emergence, and growth of seedlings in the field [1,4]. Particularly, *Brachiaria brizantha* (A. Rich.) stapf is a perennial tufted grass with great importance in the formation of improved pastures and the integrated crop–livestock system, supporting millions of animals worldwide. In Brazil, about 80% of pasture areas are occupied by different genotypes of *B. brizantha*, including the cultivars Marandu and Paiaguas [5]. Usually, the vigor of *B. brizantha* seeds is determined by tests of germination, accelerated aging, electrical conductivity, and dry mass [2]. These traditional tests are laborious and time-consuming, which hinders their use on a large scale [6].

Photonic techniques and machine learning algorithms have been extensively used to improve agricultural management in the past few years, mainly due to their high accuracy associated with their fast response, low analytical cost, simplified sample preparation, and environmentally clean techniques [7,8,9,10,11,12]. Recently, our research group demonstrated the potential application of the Fourier-transform infrared (FTIR) and Laser-induced breakdown spectroscopy (LIBS) for seed vigor classification [5,8,13]. The FTIR technique provides molecular information about the sample, while Laser-induced breakdown spectroscopy (LIBS) provides a spectrum with atomic and ionic transitions that correspond to the chemical elements of the sample. Both techniques provided satisfactory results for seed vigor classification [5,8]. The soybean seed vigor classified with 100% accuracy by using the FTIR was assigned to proteins, lipids, carbohydrates, and amides as the main molecules responsible for the discrimination [8], and similar results were observed for *B. brizantha* seeds [5]. On the other hand, 98% accuracy was achieved by using LIBS in the 350–450 nm spectral range, where transition lines assigned to Ca exhibited the main data variance. The presence of the Ca element in plants is essential for the enzymatic role that takes place during germination [13].

Thinking about on-field application, the LIBS systems can be a useful alternative, which has been proven as a versatile technique for qualitative and quantitative analysis in agricultural studies through evaluation of several samples [14] such as rice [15], maize [16], grape seeds [17], cucurbit seeds [18], coffee beans [19], and soybean seeds [13,20]. However, there are some critical issues to overcome and a need to provide a reliable method, mainly due to the lack of a consistent protocol, which may consider experimental aspects (number of samples, validation, experimental parameters, software, etc.), and machine learning algorithms limitations. For example, exclusive samples for external validation tests must be mandatory for agricultural samples, which can be easily accessed in large amounts. The results from external validation tests are the guarantee that a reliable predicting model was achieved.

An important aspect when performing the measurements is the quality of a LIBS spectrum, which is affected by experimental parameters such as laser energy, accumulated pulses, wavelength, delay time, and gate width. These parameters are fundamental in the signal optimization [21,22,23,24,25,26,27]. Experimental designs were applied to find the best conditions for LIBS experimental parameters. The design of experiments (DOE) is a systematic method used to plan and conduct experiments through the analysis and interpretation of data. It allows input variables to be manipulated to investigate their effects on the measured response variable [28] thereby improving the LIBS measurement precision [21]. Principal component analyses were applied to explore the potential separation between the groups, to evaluate outliers, and to correlate the main chemical elements with the data variance. Then, a prediction model was developed by machine learning algorithms using Phyton code. The choice of adequate models and methods used for the analysis of the spectra set is crucial to achieving a good result and fast and accurate analysis [20]. Finally, an external validation test provided 100% of accuracy for seed vigor classification. These results were mainly associated with the emission lines of Mg, Ca, and K, which yielded the greatest contribution to sample variance. Previous reports associated these ions with a fundamental enzymatic process during the germination stage.

This work aimed to discriminate the vigor quality, high and low, of brachiaria seeds from two different cultivars, Paiaguás and Mandaru, using the LIBS technique regardless of the cultivar. We overcame the main problems in agricultural studies by providing a reliable method and also a study protocol for seed vigor classification. First, we describe in detail the sample group selection: random seeds from different cultivars —the closest to a real situation in the field. Then, we used the DOE method for planning experiments. The spectra were treated with the ML tool and validated with leave-one-out cross-validation (LOO−CV) and external validation. We emphasize that no papers were found in the literature showing the efficiency of an optical technique to discriminate seed vigor considering more than one cultivar in the same set of analyses.

## 2. Materials and Methods

### 2.1. Samples and Standard Classification of the Seeds

Six different commercial batches of *B. brizantha* seeds were studied. Three of each cultivar, Paiaguás and Marandu, were previously characterized by standard procedures, according to their physiological quality, as described in previous work [5]. Two batches of Marandu seeds were classified as low vigor, while one batch was classified as high vigor, and two batches of Paiaguás seeds were classified as high vigor, while one batch was classified as low vigor. The dataset used consists of 120 *Brachiaria* seed samples in total. From the 120 seed samples, 60 present high vigor (HV), and 60 samples were low vigor (LV) (Table 1).

### 2.2. Laser-Induced Breakdown Spectroscopy System

The LIBS system is composed of a Nd:YAG pulsed laser at 532 nm Brilliant Quantel (Lumibird, Lannion, France) with maximum energy of 180 mJ, width of 4 ns, and repetition rate of 10 Hz. In addition, an echelle spectrometer Aryelle Butterfly 400 (LTB Lasertechnik Berlin GmbH, Berlin, Germany) was used to detect and select the wavelengths, and an intensified charge-coupled device (ICCD) with 1024 × 1024 pixels operated in two spectral bands (Ultraviolet (UV) at 175–330 nm and Visible (VIS) at 275–750 nm, with a resolution of 13–24 and 29–80 pm, respectively). A pulse delay generator (model 9618, Quantum Composers) was used for total temporal control of the pulsed laser and the LTB data acquisition system.

### 2.3. Optimization of Instrumental Parameters for LIBS Analyses

Instrumental parameters of LIBS influence the interaction of the laser pulse with the sample and the acquisition of the analytical signal. Thus, a 2^3^ factorial design with a central point (Table 2) was used to optimize the variables at three levels: (i) laser pulse energy (29.73, 42.29, and 54.86 mJ), (ii) delay time (0.5, 1.0, and 1.5 µs), and (iii) the analytical signal acquisition time (1, 11, and 20 µs) to obtain the LIBS spectrum. A total of 11 experiments were carried out. Each spectrum was obtained with 5 accumulated pulses, and for each experiment, 36 spectra were collected.

Approximately 200 mg of a *Brachiaria* sample was pelleted (5 tons for 1 min) and used to carry out the 11 experiments, totaling 22 pellets, 2 for each experimental run.

To identify the optimal instrumental analysis condition, the signal–background ratio (SBR) [29] was calculated for atomic (I) and ionic (II) emission lines of monitored macro and micronutrients (Al I 394.40, C I 247.856, Ca II 393.36, Cu I 324.75, Fe II 274.64, K I 766.49, Mg II 279.55, Mn II 257.61, Na I 589.59, and Si I 288.15 nm). In this strategy, each response was converted into an individual desirability value (DI), with values between 0 (undesired response and indicating low SBR value) and 1 (desired response and indicating high SBR value). The DI of each calculated emission line was combined into a single response called overall desirability (OD) [30]. In this study, the arithmetic mean was exceptionally used because some experiments resulted in DI = 0.

### 2.4. Classification Training and Prediction Methodologies

For each sample, 50 LIBS spectra were obtained in two spectral regions: UV and VIS. All data analysis steps were performed using *@Python* (3.8.5), with the Scikit-Learn machine learning library (0.23.2). LIBS spectra were subjected to a standard normal variate (SNV) preprocessing step and averaged for each sample. This procedure was performed over all raw spectra to eliminate/reduce noise and system fluctuations. After this step, the data were submitted to an outlier exclusion process using the Spectral Angle Mapper (SAM) method [31]. In this work, the average spectrum of each sample was used as a reference vector. The resulting value is a metric of the similarity between the spectra. Thus, all spectra whose SAM value in relation to the average was less than 0.90 were discarded. This process eliminated less than 5% of the spectra.

From a total of 120 samples in the dataset, 30 samples were randomly selected for the external validation step (15 HV, 15 LV) in the 2 spectral regions. The external validation process is important to avoid overfitting [13]. For both the training set and the validation set, no distinction was made between the cultivars (Marandu and Paiagua), with only seed vigor being highlighted.

The remaining 90 samples (45 HV, 45 LV) were first analyzed by Principal Component Analysis (PCA), which reduces the dimensionality of variables in the data set, transforming them into values of linearly uncorrelated variables called Principal Components (PCs). This transformation conserves the variance of the original set of variables, with a few PCs being needed to concentrate most of the data variance.

The PCA data originating from the averaged LIBS-SNV spectra were analyzed by machine learning (ML) algorithms. The classification model was performed by K-Nearest Neighbors (KNN), Linear Discriminant Analysis (LDA), Quadratic Discriminant Analysis (QDA), and Support Vector Machine (SVM) algorithms. The classification model performance was determined by the overall accuracy in the leave-one-out cross-validation (LOO−CV) test. The best classification parameters were selected by varying the number of PCs and the hyperparameter, presented in Table 3.

The supervised models were also optimized regarding the number of PCs, running from 1 to 20 PCs for each algorithm. Finally, the classification model was submitted to external validation. This procedure was applied to test the generalization bias of the model, as all validation samples are unknown to the training data set.

## 3. Results and Discussion

### Optimization of LIBS Spectra

The influence of the matrix of *Brachiaria* samples and the instrumental parameters of the LIBS system were evaluated using design of experiments (DOE) to optimize the acquisition of LIBS spectra with high SBR values for the emission lines obtained.

However, based on the ANOVA values obtained, it was not possible to obtain a regression model with significant coefficients at the level of 95% for the experimental domain evaluated. Despite not obtaining a model with good predictive capacity for all monitored elements and evaluating the calculated OD values for each experiment in Table 2, Experiment 3 presents the highest OD value (0.95) when compared to the other experiments. For this reason, in all LIBS measurements obtained in this study, 54.86 mJ were used as laser pulse energy, 0.5 µs as delay time, and 20 µs as the signal acquisition time.

**Figure 1:**
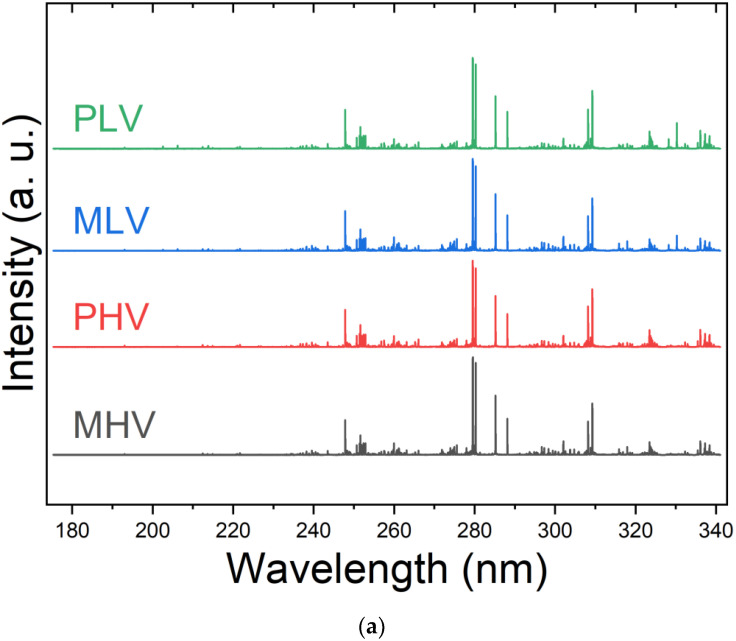

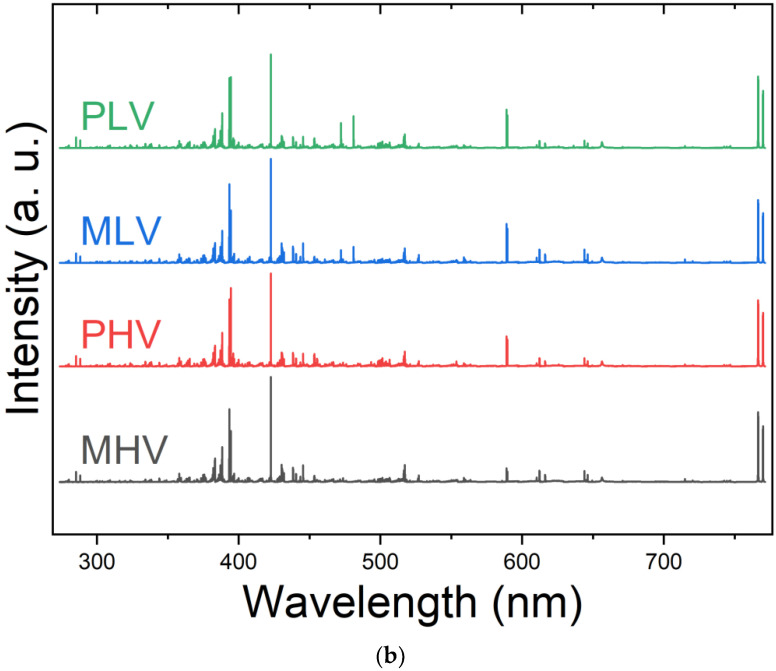
LIBS average spectra separated by cultivar and vigor classes, where MHV = Marandu High Vigor; PHV = Paiaguás High Vigor; MLV = Marandu Low Vigor; and PLV = Paiaguás Low Vigor: (**a**) UV region and (**b**) VIS region.

Figure 1 shows the normalized average spectra obtained for a *Brachiaria* sample analyzed by the LIBS system (UV and Vis region). It is observed that the typical spectrum has several element emission lines that will be evaluated in the supervised multivariate classification models.

**Figure 2:**
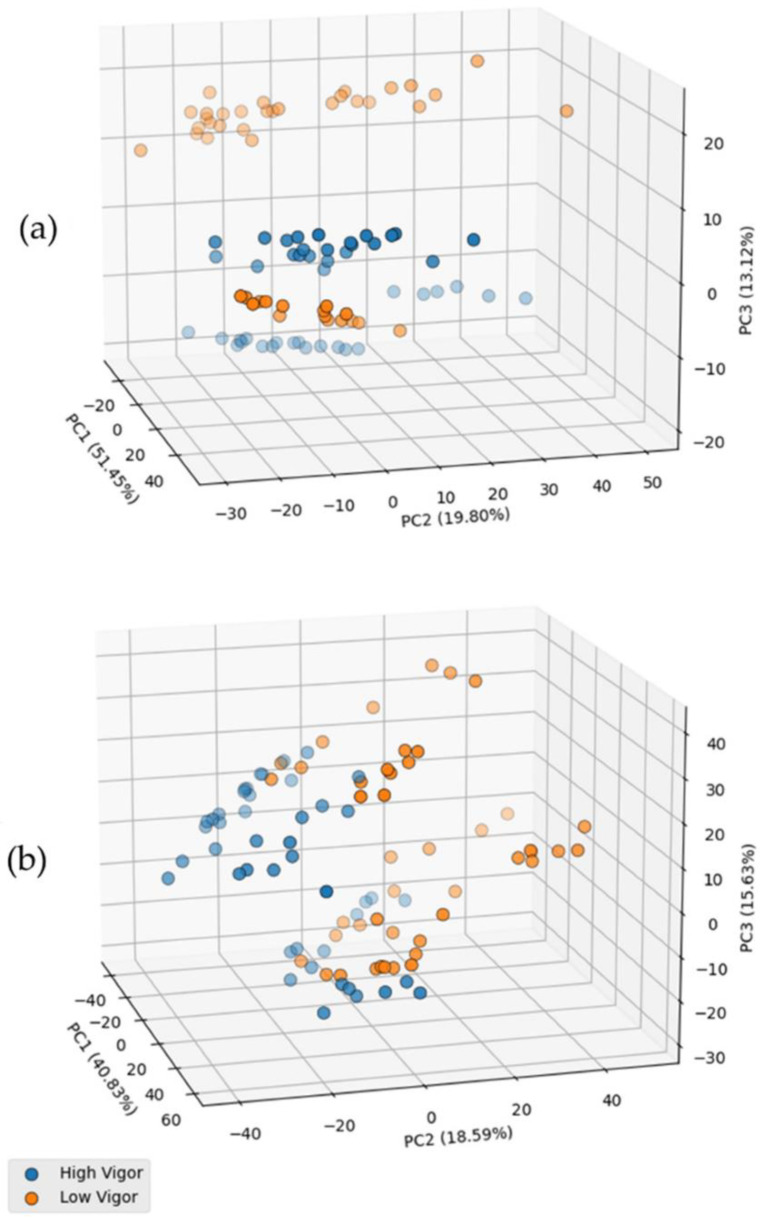
Score plots of the first three PCs: (**a**) UV region and (**b**) VIS region.

The elements C, Si, Mg, Na, Ca, Fe, and K were identified in all spectra although their relative emission intensities did not differ among the samples. In particular, the C emission line at 247.86 nm; the Fe emission line at 259.940 nm; the Mg emission line at 279.55, 280.27, and 285.213 nm; the Si emission line at 288.16 nm; the Al emission line at 309.27 and 394.40; the Ca emission line at 393.36 422.67, 442.54, 443.59, 610.27, 612.22, and 643.90; the Na emission line at 588.995; the K emission line at 766.49 and 769.90 nm; and the emission band CN at 388.35 nm were all identified. Analyzing the averaged spectra from Figure 2, it is hard to identify specific transitions that allow the seed vigor differentiation. In this sense, multivariate analysis needs to be performed.

The clustering formation in the score plot of high and low vigor groups in the UV region, Figure 2, showed the data presents a potential for group classification. The subtle difference between the high and low vigor LIBS transition spectra was better evidenced by the PC loadings (Figure S1). The data variance in the first three coordinates, PC1, PC2, and PC3, corresponds to 51.45%, 19.80%, and 13.12% in the UV region, and 40.83%, 18.59%, and 15.63% in the VIS region. As indicated by the PCA loadings of LIBS spectra (Figure S1), the highest contribution to data variance could be assigned to the emission lines of C at 247.856; Mg at 279.55, 280.27, and 285.213 nm; the Si emission line at 288.16 nm; the nAl emission line at 309.27 and 394.40; the Ca emission line at 393.36 and 422.67 nm; Na at 588.995 nm; and K at 766.54 and 769.90 nm. In particular, the emission line of Mg, Ca, and K yielded the greatest contribution to sample variance. Previous reports associated theses ions to a fundamental enzymatic process during the germination stage [13]. However, the variance among transitions needs to be analyzed with caution since they are associated with the spectra, and the statistics were performed in an unsupervised way. Analyzing the score plot in Figure 2, it is clear that the samples are being separated into two main clusters due to the difference in cultivars too. It means that the spectra present a lot of information related to the cultivars difference. In real applications, this kind of behavior is expected. Thus, it is crucial to use supervised machine learning algorithms to circumvent these issues.

For the optimization of the supervised models, a complete search around the number of PCs and parameters of greater importance in the algorithm was implemented (Table 3), and the results were shown in heat maps. The heat maps analysis is a way of visualizing the accuracy results obtained from various combinations of model parameters, making it easier to observe any trends caused by the change in these parameters. Each sample point of the heat maps was obtained by creating a model with the parameters highlighted and making a prediction on the training dataset. The accuracy value was calculated from the average of the accuracies obtained by the LOO-CV method in the internal validation model.

**Figure 3:**
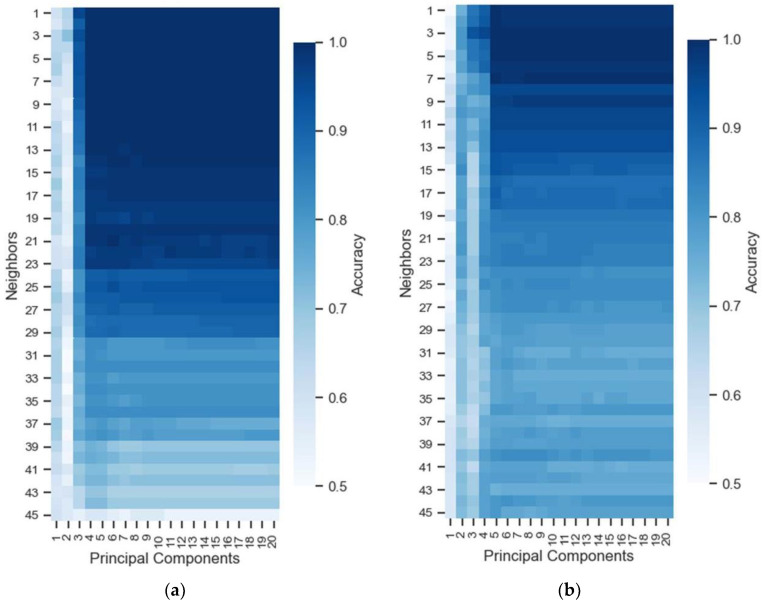
Heat map representing the accuracy values obtained in the extensive search optimizing the PC and Near Neighbors parameters in the (**a**) UV and (**b**) VIS region.

To find the best classifier for the model, a heat map of the LOO-CV test accuracies obtained by optimizing the KNN algorithm in the UV (a) and VIS (b) spectral region as shown in Figure 3 was built.

In the heat map, it is possible to observe that the accuracy of the model increased from five principal components in both spectral regions and did not benefit from a high number of close neighbors, with the best results being from three to five neighbors. This procedure was applied to all classifiers.

From the LDA algorithm, the optimized parameters were the number of PCs, from 1 to 20, and the three LDA “Solver” parameters in the Scikit-Learn library: “eigen” (eigenvalue decomposition), “lsqr” (least squares solution), and –“svd” (singular value decomposition).

**Figure 4:**
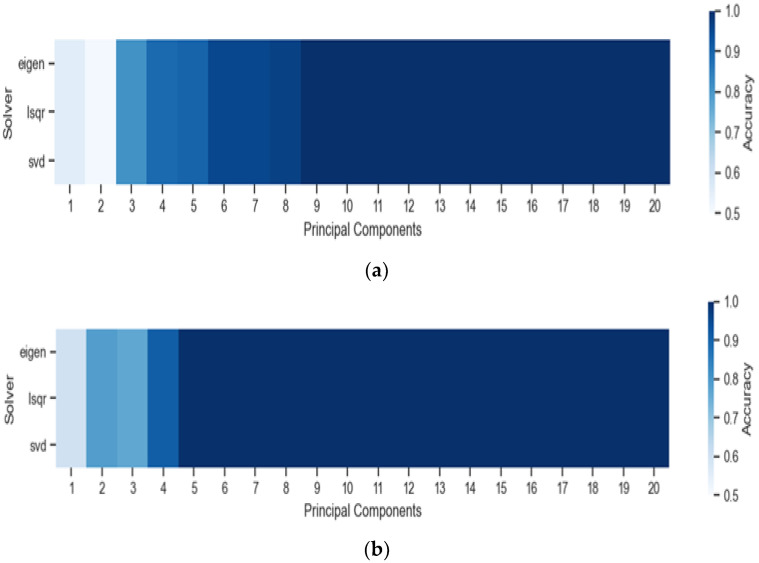
Optimization of the “PC” and “Solver” parameters in the (**a**) UV and (**b**) VIS region for the LDA algorithm.

Figure 4 presents the heat maps for the optimization of the “PC” and “Solver” parameters in the UV and VIS region for the LDA algorithm. The results were similar in both spectral regions, with the model reaching 100% accuracy from five PCs for the VIS region and nine PCs for the UV region. The “Solver” parameter did not impact the accuracy value.

**Figure 5:**
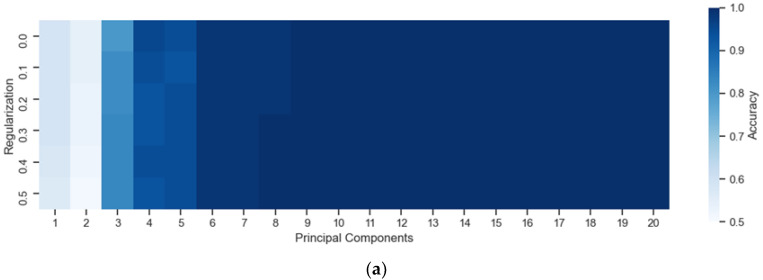

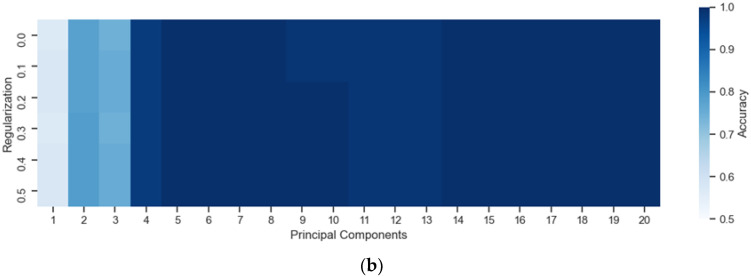
Optimization of the “Principal Components” and “Regularization” parameters in the (**a**) UV and (**b**) VIS region for the QDA algorithm.

Figure 5 presents the heat maps for the optimization of the “PC” and “regularization” parameters in the UV and VIS region using the QDA algorithm. The optimized parameters in QDA were the PCs, from 1 to 20, and the covariance matrix regularization parameter, which assumed the values: 0.0, 0.1, 0.2, 0.3, 0.4, and 0.5. It is observed in the heat maps of Figure 5, obtained from the internal validation of the models, that performance was more impacted with the number of PCs than with the regularization parameter in both spectral regions.

**Figure 6:**
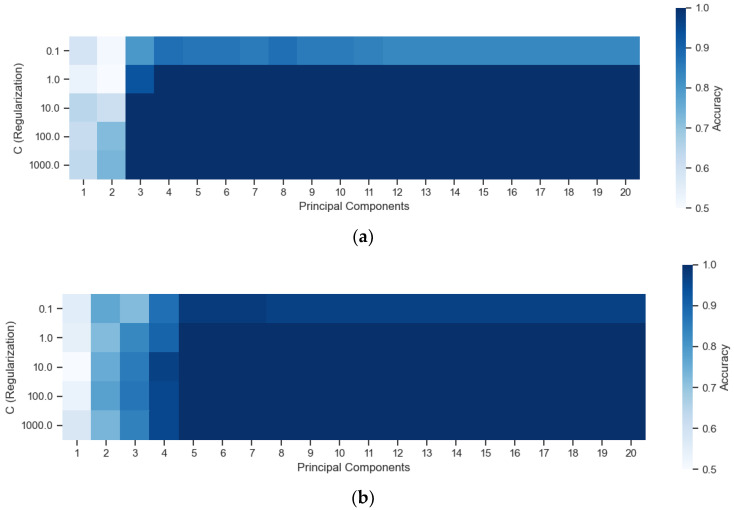
Optimization of the “Principal Components” and “C (Regularization)” parameters in the (**a**) UV region and (**b**) the VIS region for the SVM algorithm.

The last algorithm evaluated in the optimization step was the SVM, with the objective of finding the best combination of PCs, from 1 to 20, and regularization (C), at values 0.1, 10, 100, 1000, and 10,000. Figure 6 shows the optimization of the parameters “PCs” and “C (regularization)” in the UV region and the VIS region for the SVM algorithm.

As observed in the other evaluated algorithms, there is a greater dependence on accuracy with the number of PCs than with the C parameter of the SVM. Accuracies obtained in both spectral regions reached 100% for most combinations of parameters.

After selecting the most suitable classifiers for our test dataset based on the LOO-CV results presented in the heat maps, the trained algorithms performed the external validation. For the KNN method, external validation was performed with six PCs and three NN, resulting in a classification accuracy of 100% in both spectral regions. For the LDA, the accuracy obtained in external validation reached 100% with five PCs regardless of the “Solver” parameter in the VIS region. Similarly, in the UV region, the accuracy in external validation was 100% with nine PCs, also independent of the “Solver” parameter. For the QDA, the accuracy obtained reached 100% in external validation, with the best parameters being five PCs and 0.0 regularization for the VIS region and nine PCs and 0.0 regularization for the UV region. Finally, an accuracy of 100% was obtained with five PCs and regularization (C) of 1.0 in the two spectral regions evaluated.

In all algorithms, the same pattern was noticed during the optimization. The accuracy was more sensitive to the PC values than to the internal parameters of the algorithms. This was possibly due to the good separability of classes between samples in both spectral regions, as observed in the unsupervised PCA step. The external validation step showed that the parameters chosen for the algorithms do not present overfitting/underfitting and are able to generalize the trained model through an external measurement. The external validation step is fundamental in this situation to avoid bias and to demonstrate the potential for application in situations closer to reality. Thus, the classification performance in all algorithms is satisfactory both in the testing phase and in the external validation. Parameter optimization is an important step in supervised machine learning and used in conjunction with external validation ensures robust prediction models, avoiding overfitting.

## 4. Conclusions

Firstly, the parameters that influence the qualitative multi-elemental analysis of *Brachiaria brizantha* seeds LIBS were optimized using a 2^3^ factorial design, preserving the multi-element advantage of this technique. This optimization allowed describing the combined effects of the factors in the response using a smaller number of experiments. After this procedure, the supervised machine learning algorithms parameters were chosen by leave-one-out cross-validation in the test samples, and it was externally validated using a new set of data. The cross-validation tests indicated that high vigor *Brachiaria brizantha* seeds were successfully differentiated from low vigor seeds (100% accuracy) even if considering two different cultivars in the set of analyzed samples. The known fact that the LIBS technique obtains fast analysis and low analytical cost, suggests it as a potential technique for seed vigor tests of Brachiaria and, possibly, of other species. The use of DOE and chemometric methods is strongly suggested. We emphasize LIBS showed efficiency to discriminate seed vigor considering more than one cultivar in the same set of analyses.

## Figures and Tables

**Table 1:** Detail of the sample set used.

Cultivar	Vigor	Quantity
Marandu	High Vigor	20
Paiaguás	High Vigor	40
Marandu	Low Vigor	40
Paiaguás	Low Vigor	20
Total		120

**Table 2:** Matrix of 2^3^ factorial design with central point and variables evaluated for LIBS and results obtained from overall desirability (OD).

Experiment	Laser Pulse Energy		Delay Time		Signal Acquisition Time		OD
	Coded	Real (mJ)	Coded	Real (µs)	Coded	Real (µs)	
1	1	54.86	1	1.50	1	20.00	0.64
2	1	54.86	1	1.50	−1	1.00	0.63
3	1	54.86	−1	0.50	1	20.00	0.95
4	1	54.86	−1	0.50	−1	1.00	0.68
5	−1	29.73	1	1.50	1	20.00	0.64
6	−1	29.73	1	1.50	−1	1.00	0.49
7	−1	29.73	−1	0.50	1	20.00	0.33
8	−1	29.73	−1	0.50	−1	1.00	0.30
9 *	0	42.29	0	1.00	0	11.00	0.42
10 *	0	42.29	0	1.00	0	11.00	0.83
11 *	0	42.29	0	1.00	0	11.00	0.67

* Central point.

**Table 3:** Hyperparameter tested for the classification model.

Algorithm	Hyperparameter	Values	PCs
KNN	K-Neighbours	1 to 45	1 to 20
LDA	Solver	“svd”, “lsqr”, “eigen”	
QDA	Regularization	0.1, 0.2, 0.3, 0.4, 0.5	
SVM	Regularization (C)	0.1, 10, 100, 1000	

## Supplementary Materials

The following supporting information can be downloaded at: https://www.mdpi.com/article/10.3390/s22145067/s1, Figure S1: Loadings of the first three PCs. (a) Range UV e (b) Range VIS.

## References

[1] Finch-Savage W.E., Bassel G.W. (2016). Seed vigour and crop establishment: Extending performance beyond adaptation. J. Exp. Bot..

[2] Marcos Filho J. (2015). Seed vigor testing: An overview of the past, present and future perspective. Sci. Agric..

[3] TeKrony D.M., Egli D.B. (1991). Relationship of Seed Vigor to Crop Yield: A Review. Crop Sci..

[4] Rajjou L., Duval M., Gallardo K., Catusse J., Bally J., Job C., Job D. (2012). Seed Germination and Vigor. Annu. Rev. Plant Biol..

[5] Oliveira I.C., Franca T., Nicolodelli G., Morais C.P., Marangoni B., Bacchetta G., Milori D.M.B.P., Alves C.Z., Cena C. (2021). Fast and Accurate Discrimination of Brachiaria brizantha (A.Rich.) Stapf Seeds by Molecular Spectroscopy and Machine Learning. ACS Agric. Sci. Technol..

[6] Oliveira A.M.S., Nery M.C., Ribeiro K.G., Rocha A.S., Cunha P.T. (2020). Accelerated aging for evaluation of vigor in Brachiaria brizantha ‘Xaraés’ seeds. J. Seed Sci..

[7] Senesi G.S., Romano R.A., Marangoni B.S., Nicolodelli G., Villas-Boas P.R., Benites V.M., Milori D.M.B.P. (2017). Laser-Induced Breakdown Spectroscopy Associated with Multivariate Analysis Applied to Discriminate Fertilizers of Different Nature. J. Appl. Spectrosc..

[8] Larios G., Nicolodelli G., Ribeiro M., Canassa T., Reis A.R., Oliveira S.L., Alves C.Z., Marangoni B.S., Cena C. (2020). Soybean seed vigor discrimination by using infrared spectroscopy and machine learning algorithms. Anal. Methods.

[9] Belchior V., Botelho B.G., Casal S., Oliveira L.S., Franca A.S. (2020). FTIR and Chemometrics as Effective Tools in Predicting the Quality of Specialty Coffees. Food Anal. Methods.

[10] Jiang L., Mehedi Hassan M., Jiao T., Li H., Chen Q. (2021). Rapid detection of chlorpyrifos residue in rice using surface-enhanced Raman scattering coupled with chemometric algorithm. Spectrochim. Acta Part A Mol. Biomol. Spectrosc..

[11] Magalhães A.B., Senesi G.S., Ranulfi A., Massaiti T., Marangoni B.S., Nery da Silva M., Villas Boas P.R., Ferreira E., Novelli V.M., Cristofani-Yaly M. (2021). Discrimination of Genetically Very Close Accessions of Sweet Orange (Citrus sinensis L. Osbeck) by Laser-Induced Breakdown Spectroscopy (LIBS). Molecules.

[12] Nicolodelli G., Senesi G.S., Romano R.A., Cabral J., Perazzoli I.L.O., Marangoni B.S., Villas-Boas P.R., Milori D.M.B.P. (2017). Laser-induced breakdown spectroscopy of environmental and synthetic samples using non-intensified CCD: Optimization of the excitation wavelength. Appl. Phys. B Lasers Opt..

[13] Larios G.S., Nicolodelli G., Senesi G.S., Ribeiro M.C.S., Xavier A.A.P., Milori D.M.B.P., Alves C.Z., Marangoni B.S., Cena C. (2020). Laser-Induced Breakdown Spectroscopy as a Powerful Tool for Distinguishing High- and Low-Vigor Soybean Seed Lots. Food Anal. Methods.

[14] Senesi G.S., Cabral J., Menegatti C.R., Marangoni B., Nicolodelli G. (2019). Recent advances and future trends in LIBS applications to agricultural materials and their food derivatives: An overview of developments in the last decade (2010–2019). Part II. Crop plants and their food derivatives. TrAC—Trends Anal. Chem..

[15] Ribeiro M.C.S., Senesi G.S., Cabral J.S., Cena C., Marangoni B.S., Kiefer C., Nicolodelli G. (2020). Evaluation of rice varieties using LIBS and FTIR techniques associated with PCA and machine learning algorithms. Appl. Opt..

[16] Liu X., Feng X., Liu F., Peng J., He Y. (2019). Rapid Identification of Genetically Modified Maize Using Laser-Induced Breakdown Spectroscopy. Food Bioprocess Technol..

[17] He Y., Zhao Y., Zhang C., Li Y., Bao Y., Liu F. (2020). Discrimination of Grape Seeds Using Laser-Induced Breakdown Spectroscopy in Combination with Region Selection and Supervised Classification Methods. Foods.

[18] Singh J., Kumar R., Awasthi S., Singh V., Rai A.K. (2017). Laser Induced breakdown spectroscopy: A rapid tool for the identification and quantification of minerals in cucurbit seeds. Food Chem..

[19] Silva T.V., Hubinger S.Z., Gomes Neto J.A., Milori D.M.B.P., Ferreira E.J., Ferreira E.C. (2017). Potential of Laser Induced Breakdown Spectroscopy for analyzing the quality of unroasted and ground coffee. Spectrochim. Acta Part B At. Spectrosc..

[20] Li X., He Z., Liu F., Chen R. (2021). Fast Identification of Soybean Seed Varieties Using Laser-Induced Breakdown Spectroscopy Combined With Convolutional Neural Network. Front. Plant Sci..

[21] de Morais C.P., Nicolodelli G., Mitsuyuki M.C., Mounier S., Milori D.M.B.P. (2021). Optimization of laser-induced breakdown spectroscopy parameters from the design of experiments for multi-element qualitative analysis in river sediment. Spectrochim. Acta Part B At. Spectrosc..

[22] Vadillo J.M., Fernández Romero J.M., Rodríguez C., Laserna J.J. (1999). Effect of plasma shielding on laser ablation rate of pure metals at reduced pressure. Surf. Interface Anal..

[23] Aziz A., Basheer F., Sengar A., Khan S.U., Farooqi I.H. (2019). Biological wastewater treatment (anaerobic-aerobic) technologies for safe discharge of treated slaughterhouse and meat processing wastewater. Sci. Total Environ..

[24] GONDAL M., HUSSAIN T., YAMANI Z., BAIG M. (2007). The role of various binding materials for trace elemental analysis of powder samples using laser-induced breakdown spectroscopy. Talanta.

[25] Castle B.C., Talabardon K., Smith B.W., Winefordner J.D. (1998). Variables Influencing the Precision of Laser-Induced Breakdown Spectroscopy Measurements. Appl. Spectrosc..

[26] Senesi G.S., Nicolodelli G., Milori D.M.B.P., De Pascale O. (2017). Depth profile investigations of surface modifications of limestone artifacts by laser-induced breakdown spectroscopy. Environ. Earth Sci..

[27] Krüger A.L., Nicolodelli G., Villas-Boas P.R., Watanabe A., Milori D.M.B.P. (2020). Quantitative Multi-Element Analysis in Soil Using 532 nm and 1064 nm Lasers in LIBS Technique. Plasma Chem. Plasma Process..

[28] Durakovic B. (2017). Design of experiments application, concepts, examples: State of the art. Period. Eng. Nat. Sci..

[29] Castro J.P., Pereira-Filho E.R. (2016). Twelve different types of data normalization for the proposition of classification, univariate and multivariate regression models for the direct analyses of alloys by laser-induced breakdown spectroscopy (LIBS). J. Anal. At. Spectrom..

[30] Vera Candioti L., De Zan M.M., Cámara M.S., Goicoechea H.C. (2014). Experimental design and multiple response optimization. Using the desirability function in analytical methods development. Talanta.

[31] Marangoni B.S., Silva K.S.G., Nicolodelli G., Senesi G.S., Cabral J.S., Villas-Boas P.R., Silva C.S., Teixeira P.C., Nogueira A.R.A., Benites V.M. (2016). Phosphorus quantification in fertilizers using laser induced breakdown spectroscopy (LIBS): A methodology of analysis to correct physical matrix effects. Anal. Methods.

