# Supplementary figures and images for: Laser-Induced Breakdown Spectroscopy Associated with the Design of Experiments and Machine Learning for Discrimination of Brachiaria brizantha Seed Vigor
Source: Sensors (Basel). 2022 Jul 6;22(14):5067. doi: 10.3390/s22145067 (PMC9316187; doi:10.3390/s22145067)

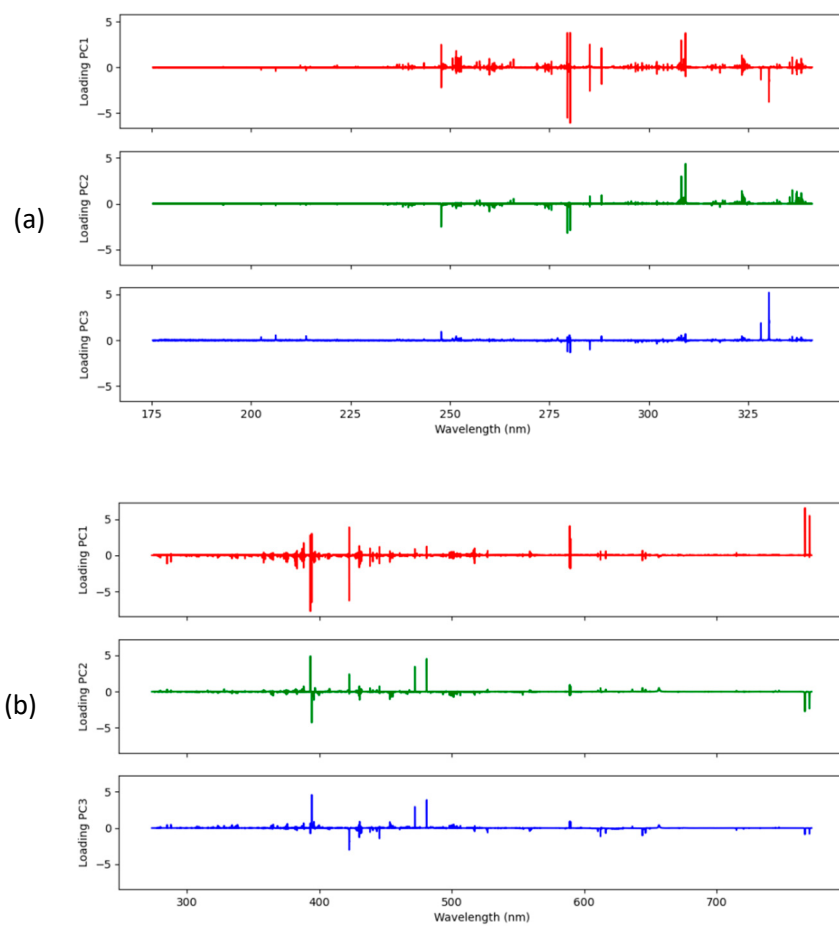

Figure S1. Loadings of the first three PCs. (a) Range UV e (b) Range VIS.

Supplement: Supplementary file 1 [file sensors-22-05067-s001.zip › sensors-1710502-supplementary.pdf]
